# Proton Pump Inhibitor Use and Survival Outcomes in Patients with Advanced Non-Small-Cell Lung Cancer Receiving Immunotherapy: A Real-World Study

**DOI:** 10.3390/medicina62071413

**Published:** 2026-07-21

**Authors:** Salih Karatlı, Seher Kaya, Selahattin Çelik, Ayşegül İlhan, Özgen Ahmet Yıldırım

**Affiliations:** Department of Medical Oncology, Etlik City Hospital, 06010 Ankara, Türkiye

**Keywords:** non-small-cell lung cancer, immune checkpoint inhibitors, proton pump inhibitors, progression-free survival, overall survival

## Abstract

*Background and Objectives*: Immune checkpoint inhibitors (ICIs) improve survival in advanced non-small-cell lung cancer (NSCLC), yet treatment responses vary. The potential influence of proton pump inhibitors (PPIs), metformin, statins, and nonsteroidal anti-inflammatory drugs (NSAIDs) on immunotherapy efficacy has gained increasing attention. This study aimed to evaluate the associations of these medications with survival outcomes. *Materials and Methods*: This retrospective cohort study included 179 patients with advanced NSCLC who received second-line nivolumab. The use of PPIs, metformin, statins, and NSAIDs was assessed within a ±30-day exposure window around nivolumab initiation. Sensitivity analyses were performed including all four studied medications. Progression-free survival (PFS) and overall survival (OS) were analyzed using the Kaplan–Meier method and Cox regression models. *Results*: Median follow-up was 19 months, and 52.5% of patients used PPIs. In univariable analysis, PPI use was significantly associated with worse PFS (HR = 2.01; *p* < 0.001), while PD-L1 ≥ 50% expression was associated with improved PFS (HR = 0.57; *p* = 0.034). In multivariable analysis, PPI use remained independently associated with worse PFS (HR = 2.22; *p* < 0.001), and male sex was associated with improved PFS (HR = 0.56; *p* = 0.013). For OS, PPI use (HR = 1.53; *p* = 0.043), ECOG ≥ 2 (HR = 2.09; *p* = 0.001), and male sex (HR = 0.53; *p* = 0.008) were significant factors in univariable analysis. In multivariable analysis, PPI use (HR = 2.01; *p* = 0.002) and ECOG ≥ 2 (HR = 2.21; *p* = 0.001) were independent adverse prognostic factors, while male sex was independently associated with improved OS (HR = 0.41; *p* = 0.001). Median PFS was 5 months in PPI users versus 9 months in non-users (*p* < 0.001); median OS was 9 versus 14 months (*p* = 0.036). The use of metformin, statins, and NSAIDs showed no significant relationship with PFS or OS. Sensitivity analyses supported an association between PPI use and worse outcomes. *Conclusions*: PPI use was associated with worse PFS and OS in patients with advanced NSCLC receiving second-line nivolumab. These findings suggest that unnecessary PPI use should be carefully reassessed during immunotherapy.

## 1. Introduction

Immune checkpoint inhibitors (ICIs), particularly the programmed cell death-1 (PD-1) inhibitor nivolumab, have significantly improved survival outcomes in patients with advanced non-small-cell lung cancer (NSCLC) who develop progression after platinum-based chemotherapy. In real-world data, responses to ICI therapy show considerable heterogeneity, and it is suggested that, beyond tumor biology, clinical factors such as concomitant medications may also influence treatment efficacy [1,2,3,4,5].

Proton pump inhibitors (PPIs) are among the most commonly used medications in cancer patients, and increasing evidence in recent years suggests that these agents may negatively affect the efficacy of ICIs. This effect is thought to occur through mechanisms such as alterations in the gut microbiota, reduced microbial diversity, and suppression of antitumor immune responses [3,6]. Indeed, several meta-analyses have demonstrated that PPI use during ICI therapy is associated with poorer progression-free survival (PFS) and overall survival (OS). In addition to PPIs, other commonly used medications such as metformin, statins, and nonsteroidal anti-inflammatory drugs (NSAIDs) have also been proposed to influence immunotherapy efficacy through immune and metabolic pathways. However, the clinical evidence regarding these agents remains limited and inconsistent [7,8,9,10,11].

Although previous cohort studies and meta-analyses have evaluated the association between PPI use and immune checkpoint inhibitor outcomes, the present study provides additional real-world evidence from a relatively homogeneous cohort treated exclusively with second-line nivolumab and simultaneously examines the associations of PPIs, metformin, statins, and NSAIDs with survival outcomes within the same population.

This retrospective cohort study investigated the relationship between PPI exposure and PFS and OS in patients with advanced NSCLC treated with second-line nivolumab, while also assessing whether metformin, statins, and NSAIDs had independent associations with survival after adjustment for established clinical factors.

## 2. Materials and Methods

### 2.1. Study Design and Ethical Approval

This study was a single-center, retrospective cohort study conducted at the Medical Oncology Clinic of Ankara Etlik City Hospital. Patients who were admitted to our center between 1 October 2022 and 1 April 2026 with a diagnosis of advanced NSCLC and who had received or were receiving second-line nivolumab treatment following progression after platinum-based chemotherapy were included. Nivolumab treatment was continued until disease progression, unacceptable toxicity, death, or treatment discontinuation for clinical reasons; therefore, treatment duration varied among patients.

In the previous study, 88 patients with recurrent or stage IV NSCLC treated with second-line nivolumab were analyzed to determine whether prior mRNA COVID-19 vaccination was associated with PFS2 [12]. Because of systemic corticosteroid use, incomplete follow-up data, and additional exclusion criteria applied in the present study, only 70 patients from the previous cohort were included in the current analysis. The present study included a larger cohort of 179 patients and examined the associations of PPI, metformin, statin, and NSAID use with PFS and OS. Although both studies were conducted at the same center in a similar treatment population, their research hypotheses, exposure variables, endpoints, and statistical analyses were distinct. All statistical and survival analyses were performed de novo in the current cohort, and no analyses or results from the previous publication were reused.

The study was performed in compliance with the Declaration of Helsinki and received approval from the Scientific Research Ethics Committee of Ankara Etlik City Hospital (Decision No: AEŞH-BADEK1-2026-313; Date: 8 April 2026). Due to the retrospective design and use of anonymized data, the requirement for written informed consent was waived by the Scientific Research Ethics Committee of Ankara Etlik City Hospital.

### 2.2. Patient Selection and Criteria

Patients aged ≥ 18 years with histopathologically confirmed advanced NSCLC who received immunotherapy in the second-line setting during the study period were screened for eligibility. Patients who met the predefined eligibility criteria and received second-line nivolumab were included in the final analysis.

#### 2.2.1. Inclusion Criteria

• Histopathologically confirmed NSCLC.
• Progression after platinum-based chemotherapy.
• Receipt of or ongoing second-line nivolumab treatment.
• Age ≥ 18 years.

#### 2.2.2. Exclusion Criteria

• The use of ICIs other than nivolumab.
• Missing follow-up data or unknown date of progression/death.
• Active second primary malignancy.
• Systemic corticosteroid use.

Systemic corticosteroid exposure was defined as ongoing oral or parenteral corticosteroid treatment at the time of nivolumab initiation. Patients receiving systemic corticosteroids at nivolumab initiation were excluded regardless of dose or clinical indication. Because of inconsistent documentation in the retrospective records, a formal dose threshold could not be reliably applied. Previous intermittent systemic corticosteroid use was not considered an exclusion criterion if treatment had been discontinued before nivolumab initiation. Inhaled, topical, and locally administered corticosteroids were not classified as systemic exposure and were therefore not exclusionary.

This exclusion was applied because systemic corticosteroids exert potent immunosuppressive effects, including inhibition of T-cell function, and may diminish the efficacy of nivolumab. Baseline exposure to systemic corticosteroids has likewise been linked to poorer survival among patients treated with immune checkpoint inhibitors. This criterion was therefore implemented to reduce potential confounding and to allow for a clearer evaluation of the association between concomitant medication use and survival outcomes.

#### 2.2.3. Definition of Drug Exposure

PPI use was defined as documented regular use for at least four weeks within the ±30-day window around nivolumab initiation, extending from 30 days before to 30 days after treatment initiation. Regular use was determined from the hospital electronic medical records, including active medication lists and available prescription records. The same exposure definition was applied to metformin, statins, and NSAIDs. Patients who initiated PPI treatment only after nivolumab initiation were included in the primary analysis according to the predefined exposure window and were separately identified for a timing-related sensitivity analysis. Patients who experienced early progression or death were classified according to their documented exposure status in the available medical records, and these early outcomes did not alter exposure classification. Detailed information regarding the specific agent, dose, exact duration, treatment indication, adherence, continuation after nivolumab initiation, possible over-the-counter use, and a reliable distinction between short-term and chronic exposure was not consistently available in the retrospective records.

Antibiotic use, which is known to have a profound impact on the gut microbiota and immunotherapy outcomes, was not included in the analysis. This was primarily due to the retrospective design of the study, which limited the accurate assessment of antibiotic exposure in terms of timing, duration, indication, and cumulative dose. In particular, the distinction between short-term and chronic antibiotic use could not be reliably established, and incomplete or inconsistent medical records further restricted precise classification. Therefore, to avoid potential misclassification bias, antibiotic use was not incorporated into the analytical models.

### 2.3. Data Collection

Demographic and clinical data were retrospectively obtained from the hospital electronic record system.

The recorded variables included the following:

• Age and sex.
• Smoking status (never, current, former).
• Histological subtype (adenocarcinoma or others).
• Eastern Cooperative Oncology Group (ECOG) performance status.
• Programmed death-ligand 1 (PD-L1) expression level (<1%, 1–49%, ≥50%).
• Use of PPIs, metformin, statins, and NSAIDs.
• Dates of progression and death.

PD-L1 expression was evaluated locally in the pathology laboratory of our institution by immunohistochemistry using the VENTANA PD-L1 (SP263) Rabbit Monoclonal Primary Antibody on the Ventana BenchMark ULTRA platform (Ventana Medical Systems, Inc., Tucson, AZ, USA) as part of the routine clinical diagnostic work-up. Testing was performed on available archival tumor tissue obtained before nivolumab initiation, and no additional biopsy was performed specifically for this study. The tumor proportion score (TPS) was used for scoring, and the results were categorized as <1%, 1–49%, and ≥50%.

### 2.4. Endpoints

PFS was defined as the time from initiation of nivolumab treatment to radiological disease progression or death from any cause, whichever occurred first. Tumor response and disease progression were evaluated according to the Response Evaluation Criteria in Solid Tumors, version 1.1 (RECIST 1.1), using radiological assessments performed during routine clinical follow-up. The timing of imaging assessments followed routine clinical practice and was not dictated by a prespecified study protocol. In cases with suspected pseudoprogression or equivocal radiological findings, subsequent imaging and the treating physician’s clinical assessment were considered before confirming progression. Patients without progression or death were censored at the date of the last radiological assessment.

OS was defined as the time from initiation of nivolumab treatment to death from any cause. Patients without a recorded death were censored at the date of last follow-up.

### 2.5. Statistical Analysis

Continuous variables were expressed as the median (minimum–maximum), and categorical variables as number and percentage.

Comparisons between groups were performed using the Mann–Whitney U test for continuous variables and the chi-square test for categorical variables.

Survival analyses were conducted using the Kaplan–Meier method, and differences between groups were evaluated with the log-rank test.

Univariable and multivariable Cox regression analyses were performed to identify prognostic factors. Variables with *p* < 0.10 in univariable analysis were included in the multivariable model. Additionally, a sensitivity multivariable Cox regression model including all four studied medications (NSAIDs, metformin, statins, and PPIs) was constructed regardless of univariable significance. To assess potential timing-related bias, an additional sensitivity analysis was performed after excluding the eight patients who initiated PPI treatment only after nivolumab initiation.

Effect estimates were presented as HRs together with their corresponding 95% confidence intervals (CIs). All tests were two-sided, and *p* < 0.05 was considered statistically significant.

Statistical analyses were performed using IBM SPSS Statistics for Windows, version 26.0 (IBM Corp., Armonk, NY, USA).

## 3. Results

### 3.1. Patient Characteristics

**Figure 1:**
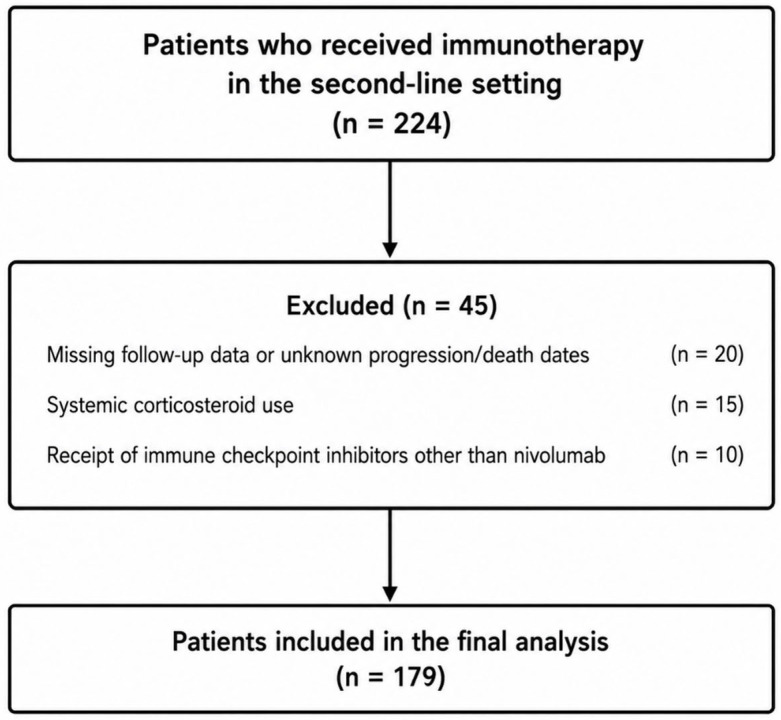
Flow diagram of patient selection, exclusion, and inclusion in final analysis.

A total of 224 patients receiving immunotherapy in the second-line setting were assessed for eligibility. Of these, 45 were excluded because of missing follow-up data or unknown progression or death dates (*n* = 20), systemic corticosteroid use (*n* = 15), or receipt of an ICI other than nivolumab (*n* = 10). Consequently, 179 patients receiving second-line nivolumab were included in the final analysis (Figure 1).

The median follow-up duration in the final cohort was 19 months (range, 1–41 months). At the data cut-off, 124 progression or death events were recorded for the PFS analysis, and 95 deaths were recorded for the OS analysis. The median age was 66 years (36–88), and 82.7% of the patients were male.

In terms of histological subtypes, 60.3% of patients had adenocarcinoma and 39.7% had squamous cell carcinoma. According to performance status, the majority of patients were in the ECOG 0–1 group (82.1%), while 17.9% had ECOG ≥ 2.

PD-L1 expression was <1% in 60.3% of patients, 1–49% in 17.9%, and ≥50% in 21.8%. Regarding smoking status, 70.9% were current smokers, 20.1% were former smokers, and 8.9% had never smoked.

Among the studied medications, 52.5% of patients were using PPIs, 54.7% were using NSAIDs, 20.7% were using statins, and 18.4% were using metformin (Table 1).

Table 2 summarizes the baseline characteristics stratified by PPI exposure. Most baseline clinical and pathological characteristics were comparable between PPI users and non-users. Male sex was more frequent among PPI users than among non-users (89.4% vs. 75.3%, *p* = 0.013). In contrast, age, histology, ECOG performance status, PD-L1 expression, smoking status, metformin use, statin use, NSAID use, metastatic sites, and the number of metastatic sites did not differ significantly between groups.

### 3.2. PFS Analysis

In univariable Cox regression analysis, PPI use was significantly associated with shorter PFS (HR = 2.01; 95% CI: 1.39–2.89; *p* < 0.001). Additionally, patients with PD-L1 ≥ 50% expression had significantly better PFS compared with those with PD-L1 < 1% (HR = 0.57; 95% CI: 0.34–0.96; *p* = 0.034). Male sex showed a borderline association with improved PFS compared with female sex (HR = 0.68; 95% CI: 0.44–1.05; *p* = 0.080). Other variables, including age, histological subtype, ECOG performance status, smoking status, and the use of metformin, statins, and NSAIDs, were not significantly associated with PFS (all *p* > 0.05) (Table 3).

In the multivariable analysis including variables with *p* < 0.10 in univariable analysis, PPI use remained independently associated with worse PFS (HR = 2.22; 95% CI: 1.52–3.24; *p* < 0.001). Male sex was independently associated with improved PFS compared with female sex (HR = 0.56; 95% CI: 0.36–0.89; *p* = 0.013). PD-L1 ≥ 50% expression showed a trend toward improved PFS but did not reach statistical significance in the multivariable model (HR = 0.61; 95% CI: 0.36–1.01; *p* = 0.056) (Table 3).

In a sensitivity multivariable model including all four studied medications, PPI use remained independently associated with shorter PFS (HR = 2.40; 95% CI: 1.61–3.60; *p* < 0.001). Male sex was associated with improved PFS (HR = 0.59; 95% CI: 0.37–0.93; *p* = 0.023), and PD-L1 ≥ 50% expression also remained associated with improved PFS (HR = 0.58; 95% CI: 0.34–0.98; *p* = 0.042). NSAID, metformin, and statin use were not significantly associated with PFS (Supplementary Table S1). After excluding the eight patients who initiated PPI treatment only after nivolumab initiation, PPI use remained independently associated with shorter PFS (HR = 2.47; 95% CI: 1.65–3.71; *p* < 0.001). The findings were consistent with the primary analysis (Supplementary Table S3).

**Figure 2:**
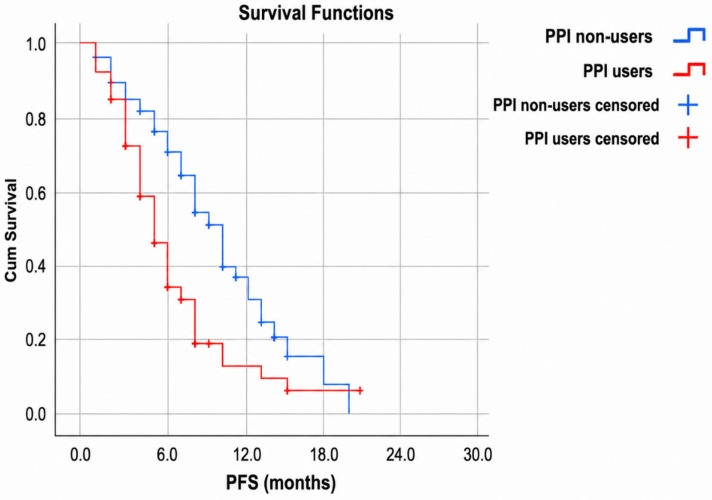
Kaplan–Meier curves for PFS according to PPI use. Patients receiving PPIs had significantly shorter PFS compared to non-users (median PFS: 5 [4.2–5.8] vs. 9 [7.6–10.4] months; log-rank *p* < 0.001). Abbreviations: PFS, progression-free survival; PPI, proton pump inhibitor.

Kaplan–Meier estimates indicated a significant reduction in PFS among patients exposed to PPIs. The median PFS was 5 months (95% CI: 4.18–5.81) in the PPI group and 9 months (95% CI: 7.62–10.38) in the non-PPI group (log-rank *p* < 0.001) (Figure 2). Median PFS was also evaluated according to metformin, statin, and NSAID use, and no significant differences were observed between users and non-users of these medications).

### 3.3. OS Analysis

In univariable analysis, ECOG ≥ 2 was significantly associated with worse OS (HR = 2.09; 95% CI: 1.33–3.28; *p* = 0.001). PPI use was also associated with worse OS (HR = 1.53; 95% CI: 1.01–2.30; *p* = 0.043). In contrast, male sex was significantly associated with improved OS compared with female sex (HR = 0.53; 95% CI: 0.33–0.85; *p* = 0.008).

Although PD-L1 ≥ 50% expression showed a trend toward improved survival, it did not reach statistical significance (HR = 0.56; 95% CI: 0.31–1.03; *p* = 0.062). Age, histological subtype, smoking status, and the use of metformin, statins, and NSAIDs were not significantly associated with OS (all *p* > 0.05) (Table 4).

In multivariable analysis, PPI use remained independently associated with worse OS (HR = 2.01; 95% CI: 1.28–3.15; *p* = 0.002). ECOG ≥ 2 performance status also remained an independent adverse prognostic factor for OS (HR = 2.21; 95% CI: 1.39–3.51; *p* = 0.001). In contrast, male sex was independently associated with improved OS compared with female sex (HR = 0.41; 95% CI: 0.25–0.69; *p* = 0.001). PD-L1 expression was not an independent predictor of OS in the multivariable analysis (Table 4).

In the sensitivity analysis for OS, PPI use (HR = 1.97; 95% CI: 1.23–3.16; *p* = 0.005) and ECOG ≥ 2 (HR = 2.14; 95% CI: 1.34–3.42; *p* = 0.001) remained independent adverse prognostic factors. Male sex was independently associated with improved OS (HR = 0.41; 95% CI: 0.24–0.68; *p* = 0.001), whereas NSAID, metformin, and statin use were not significantly associated with OS (Supplementary Table S2). After excluding the eight patients who initiated PPI treatment only after nivolumab initiation, PPI use remained independently associated with worse OS (HR = 1.91; 95% CI: 1.20–3.05; *p* = 0.007). The findings were consistent with the primary analysis (Supplementary Table S4).

**Figure 3:**
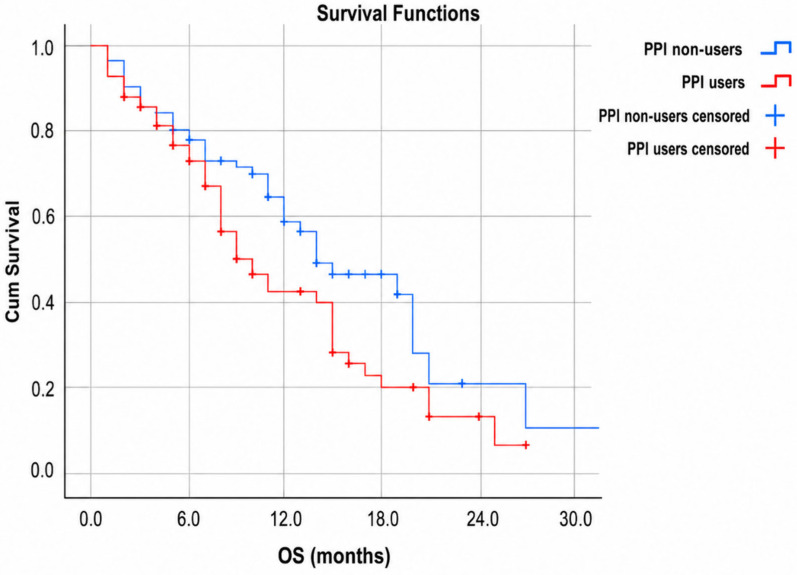
Kaplan–Meier curves for OS according to PPI use. Patients receiving PPIs had significantly shorter OS compared to non-users (median OS: 9 [6.5–11.5] vs. 14 [8.5–19.5] months; log-rank *p* = 0.036). Abbreviations: OS, overall survival; PPI, proton pump inhibitor.

Kaplan–Meier analysis demonstrated that median OS was shorter in patients using PPIs (9 months vs. 14 months; log-rank *p* = 0.036) (Figure 3; Table 5). Median OS was also evaluated according to metformin, statin, and NSAID use, and no significant differences were observed between users and non-users of these medications (Table 5).

## 4. Discussion

In this study, the associations of PPI, metformin, statin, and NSAID use with survival outcomes were evaluated in patients with advanced NSCLC receiving second-line nivolumab. Our findings indicate that PPI use is associated with worse clinical outcomes in patients undergoing immunotherapy, whereas the effects of other commonly used medications such as metformin, statins, and NSAIDs appear to be more limited and heterogeneous.

The negative association between PPI use and cancer-related outcomes has been increasingly reported in recent years. Recent systematic reviews and meta-analyses have associated long-term PPI exposure with a modestly increased incidence of certain malignancies, particularly gastric and other gastrointestinal cancers, although the magnitude and consistency of these associations vary across cancer types and study designs. In patients receiving ICIs, meta-analyses and large cohort studies have similarly shown that PPI exposure is associated with shorter PFS and OS. However, because most of the available evidence is observational, these findings should not be interpreted as establishing a direct causal relationship. Confounding by indication, reverse causation, underlying comorbidities, and differences in disease burden may partly explain the poorer outcomes observed among PPI users. Nevertheless, a potential biological effect remains plausible, particularly through alterations in the gut microbiota, which plays a key role in regulating innate and adaptive immunity and may directly influence the response to ICIs [4,13,14,15,16,17,18].

Several mechanisms have been proposed to explain how PPIs may disrupt this process. Gastric acid suppression may lead to reduced microbial diversity and dysbiosis, resulting in the loss of bacterial species that support immune function. In particular, a decrease in bacteria such as *Akkermansia muciniphila*, which have been associated with response to immunotherapy, may impair T-cell activation and antitumor immunity. Additionally, PPI use may alter microbiota–immune cell interactions, increase immunosuppressive cell populations, and reduce the efficacy of PD-1 blockade. These microbiome-related alterations may also influence sensitivity to immune checkpoint inhibition and potentially affect the occurrence or pattern of immune-related adverse events [19,20,21].

Consistent with previous reports, PPI use was independently associated with shorter PFS and OS in our cohort, even after adjustment for established clinical prognostic factors, including ECOG performance status and PD-L1 expression. Nevertheless, this observational association should not be interpreted as evidence of causality. PPI use may serve as a surrogate marker for adverse underlying clinical characteristics, such as greater symptom burden, comorbidity, poorer general condition, or more aggressive disease, which may themselves contribute to reduced survival. Importantly, baseline metastatic involvement, including liver, brain, bone, and other distant sites, was comparable across the PPI-exposed and unexposed groups. Furthermore, patients receiving systemic corticosteroids were systematically excluded to reduce potential confounding related to steroid-associated PPI use and the adverse prognostic impact of corticosteroid requirement. However, residual confounding due to unmeasured factors, such as symptom burden, disease aggressiveness, and other supportive care requirements, cannot be completely excluded. Accordingly, the current results do not establish that PPIs directly worsen tumor progression or reduce immunotherapy effectiveness. Prospective studies are needed to better define the nature of this relationship.

Regarding metformin, there is considerable mechanistic evidence suggesting that this agent may enhance immunotherapy response. Metformin can activate the AMP-activated protein kinase (AMPK) pathway, reprogram tumor cell metabolism, and contribute to reduced PD-L1 expression [22,23]. It has also been shown to enhance immune response by preserving CD8+ T-cell function in hypoxic tumor microenvironments and reducing T-cell exhaustion [24,25]. However, clinical studies evaluating the impact of metformin on immunotherapy outcomes have yielded inconsistent results, and a survival benefit has not been consistently demonstrated [26,27]. In our study, no significant association was observed between metformin use and PFS or OS, which is consistent with the heterogeneous findings in the literature. The potential benefit of metformin may depend on patient subgroups, metabolic conditions (e.g., insulin resistance), and drug-related factors such as dose and duration. Additionally, the relatively low rate of metformin use in our cohort and the retrospective design may have contributed to the lack of a significant effect.

With regard to statins, previous studies have suggested potential beneficial effects on immunotherapy outcomes through both mechanistic and clinical evidence. Statins may inhibit tumor cell proliferation by targeting the mevalonate pathway, enhance T-cell activation, and shift macrophages from an immunosuppressive M2 phenotype toward an antitumor M1 phenotype; however, clinical data remain heterogeneous [28,29,30]. Our analysis did not identify a significant relationship between statin use and survival outcomes, which contrasts with some reports in the literature. Possible explanations include the limited sample size, differences between statin types (lipophilic vs. hydrophilic), and heterogeneity in dose and duration.

Regarding NSAID use, there is strong evidence suggesting that aspirin, in particular, may enhance immunotherapy efficacy. This effect is thought to be mediated through inhibition of cyclooxygenase (COX), leading to reduced prostaglandin E2 (PGE2) levels and suppression of the immunosuppressive tumor microenvironment. Additionally, NSAID use may enhance T-cell responses by reducing neutrophil-mediated immune suppression [31,32]. By contrast, our analysis did not demonstrate a significant relationship between NSAID exposure and survival, a finding that only partly aligns with previous reports. The most likely explanation is that aspirin and other NSAIDs were analyzed as a single group, preventing the evaluation of drug-specific effects.

Evidence from multiple studies suggests that elevated PD-L1 expression is associated with better survival outcomes, especially in the first-line setting. However, this relationship appears to be less consistent in later lines of therapy and real-world cohorts. In our cohort of patients receiving second-line nivolumab, PD-L1 ≥ 50% expression was associated with improved PFS in univariable analysis and showed a trend toward improved PFS in the primary multivariable model, although it did not reach statistical significance. In contrast, PD-L1 expression was not independently associated with OS. This finding may be explained by the influence of subsequent lines of therapy and the stronger prognostic impact of clinical factors such as ECOG performance status, which may attenuate the independent effect of PD-L1 on overall survival, particularly in later-line treatment settings. The modest sample size may also have limited the statistical power to identify an independent association, particularly with OS [33,34,35].

ECOG performance status is well established as a strong prognostic factor for OS, whereas its association with PFS appears to be more variable in patients receiving immunotherapy. In our study, ECOG ≥ 2 was not significantly associated with PFS, either in univariable analysis or in the primary multivariable model. This may be related to the heterogeneous response patterns observed with ICIs, including delayed responses and atypical disease dynamics, which may reduce the direct influence of baseline performance status on early progression. In addition, the relatively low proportion of patients with ECOG ≥ 2 in our cohort (17.9%) may have limited the statistical power to detect a significant association with PFS. In contrast, ECOG ≥ 2 remained an independent adverse prognostic factor for OS, supporting the view that patient-related clinical factors have a stronger impact on long-term survival outcomes in real-world later-line treatment settings [33,34,35].

In our study, male sex was independently associated with improved PFS and OS in multivariable analyses. However, this finding should be interpreted with caution. Given the high proportion of male patients in our cohort, the relatively small female subgroup, and the potential association between male sex and smoking history, this result may reflect smoking-related tumor biology and cohort composition rather than a direct effect of sex itself. Smoking-associated NSCLC may have higher tumor mutational burden, which has been linked to greater responsiveness to ICIs [36]. Therefore, the favorable survival outcomes observed in male patients may partly be explained by smoking-related immunogenicity. However, the retrospective nature of the study and the absence of quantitative smoking exposure measures, including pack-year data, prevented a comprehensive evaluation of this hypothesis. Thus, male sex should be interpreted as a clinical marker potentially associated with smoking history and tumor biology rather than as a direct causal determinant of improved survival.

This study has several important limitations. First, its retrospective, single-center design increases the risk of selection bias and may limit the generalizability of the findings. Detailed medication-related information, including the specific agent, dose, exact duration, timing, indication, and adherence, could not be comprehensively assessed. Regular medication use was determined from available electronic medical records and prescription documentation; however, pharmacy refill data, direct adherence measures, and possible over-the-counter use were not systematically available, and actual adherence could therefore not be independently verified. Although baseline metastatic sites and the number of metastatic sites were available and compared between PPI users and non-users, more detailed measures of disease burden, including lesion count, metastatic volume, and longitudinal changes in metastatic disease, could not be evaluated. Other potentially relevant clinical factors, such as comorbidity burden, diabetes status, glycemic control, body mass index, renal function, symptom burden, prior treatment details, supportive medication requirements, inflammatory laboratory markers, quantitative smoking exposure, and the clinical indication for PPI prescription, were unavailable or incompletely documented. Subsequent treatments after progression on nivolumab were heterogeneous and were not analyzed according to specific regimen, as the present study was not designed to compare post-progression treatment strategies. Nevertheless, their potential influence on OS cannot be completely excluded. H2-receptor antagonist use was not evaluated; therefore, a direct comparison with PPIs could not be performed. Similarly, antibiotic exposure, a major potential confounder known to affect the gut microbiome and immunotherapy efficacy, could not be reliably assessed because of incomplete and heterogeneous documentation regarding timing, duration, indication, and cumulative exposure. Thus, unmeasured confounding from antibiotic exposure may still be present and could have contributed, at least in part, to the observed relationship between PPI use and survival. In addition, a formal residual-based assessment of the proportional hazard assumption was not performed. The associations observed between PPI exposure and survival should therefore be interpreted in light of these limitations.

The study also has several strengths. The inclusion of a relatively homogeneous population treated exclusively with second-line nivolumab reduced treatment-related heterogeneity. In addition, the simultaneous evaluation of four commonly used concomitant medication classes—PPIs, metformin, statins, and NSAIDs—within the same cohort provided a broader assessment of their associations with survival outcomes. The use of multivariable analyses and a timing-related sensitivity analysis excluding patients who initiated PPI therapy after nivolumab initiation further strengthened the robustness and interpretability of the findings.

Importantly, these findings should not be interpreted as supporting the discontinuation of PPIs in patients with clear and appropriate clinical indications. Rather, they highlight the need to avoid potentially unnecessary PPI use and to further investigate this association in prospective studies.

## 5. Conclusions

This study suggests that PPI use is associated with worse survival outcomes in patients with advanced NSCLC receiving immunotherapy. However, the observational design and the possibility of residual confounding preclude causal interpretation. PPI use may serve as a clinical marker of poorer prognosis rather than a directly modifiable prognostic factor. Prospective studies are warranted to determine whether PPI exposure directly influences immunotherapy outcomes.

## Figures and Tables

**Table 1:** Baseline Characteristics of Study Population (*n* = 179).

Variable	*n* (%)
Age, median (range)	66 (36–88)
Sex	
Female	31 (17.3)
Male	148 (82.7)
Histology	
Adenocarcinoma	108 (60.3)
SCC	71 (39.7)
ECOG Performance Status	
0–1	147 (82.1)
≥2	32 (17.9)
PD-L1 Expression	
<1%	108 (60.3)
1–49%	32 (17.9)
≥50%	39 (21.8)
Smoking Status	
Never	16 (8.9)
Current smoker	127 (70.9)
Former smoker	36 (20.1)
Studied Medications	
PPI use	94 (52.5)
Metformin use	33 (18.4)
Statin use	37 (20.7)
NSAID use	98 (54.7)
Median follow-up, months (min–max)	19 (1–41)

Abbreviations: ECOG, Eastern Cooperative Oncology Group; PD-L1, programmed death-ligand 1; SCC, squamous cell carcinoma; PPI, proton pump inhibitor; NSAID, nonsteroidal anti-inflammatory drug. Note: Values are presented as number (percentage) unless otherwise indicated.

**Table 2:** Comparison of Baseline Clinical and Pathological Characteristics According to PPI Exposure.

Variable	No PPI Use *n* (%)	PPI Use *n* (%)	*p*-Value
Age, median (range)	66 (50–81)	67 (36–88)	0.928
Sex			0.013
Female	21 (24.7)	10 (10.6)	
Male	64 (75.3)	84 (89.4)	
Histology			0.315
Adenocarcinoma	48 (56.5)	60 (63.8)	
SCC	37 (43.5)	34 (36.2)	
ECOG performance status			0.137
ECOG 0–1	66 (77.6)	81 (86.2)	
ECOG ≥2	19 (22.4)	13 (13.8)	
PD-L1 expression			0.259
<1%	54 (63.5)	54 (57.4)	
1–49%	11 (12.9)	21 (22.3)	
≥50%	20 (23.5)	19 (20.2)	
Smoking status			0.296
Never smoker	10 (11.8)	6 (6.4)	
Current smoker	56 (65.9)	71 (75.5)	
Former smoker	19 (22.4)	17 (18.1)	
Metformin use			0.796
No	70 (82.4)	76 (80.9)	
Yes	15 (17.6)	18 (19.1)	
Statin use			0.562
No	69 (81.2)	73 (77.7)	
Yes	16 (18.8)	21 (22.3)	
NSAID use			0.288
No	42 (49.4)	39 (41.5)	
Yes	43 (50.6)	55 (58.5)	
Metastatic sites			
Liver metastasis	10 (11.8)	12 (12.8)	0.839
Adrenal metastasis	16 (18.8)	18 (19.1)	0.956
Brain/CNS metastasis	13 (15.3)	17 (18.1)	0.618
Bone metastasis	27 (31.8)	30 (31.9)	0.983
Pleural metastasis	21 (24.7)	22 (23.4)	0.839
Contralateral lung metastasis	11 (12.9)	17 (18.1)	0.344
Other metastatic sites	24 (28.2)	21 (22.3)	0.364
Number of metastatic sites			0.941
Single metastatic site	52 (61.2)	57 (60.6)	
≥2 metastatic sites	33 (38.8)	37 (39.4)	

Abbreviations: SCC, squamous cell carcinoma; ECOG, Eastern Cooperative Oncology Group; PD-L1, programmed death-ligand 1; PPI, proton pump inhibitor; NSAID, nonsteroidal anti-inflammatory drug; CNS, central nervous system. Note: Values are presented as number (percentage) unless otherwise indicated. Age is presented as median (range). *p*-values were calculated using the chi-square test for categorical variables and the Mann–Whitney U test for age. Other metastatic sites included non-regional lymph nodes, abdominal lymph nodes, scalene/cervical lymph nodes, peritoneal, gastrointestinal/colon, and other rare metastatic sites.

**Table 3:** Cox Regression Analysis for Progression-Free Survival (PFS).

Variable	Univariable HR (95% CI)	*p*-Value	Multivariable HR (95% CI)	*p*-Value
Age, per 1-year increase	0.99 (0.97–1.01)	0.350	–	–
Male vs. female	0.68 (0.44–1.05)	0.080	0.56 (0.36–0.89)	0.013
Histology (SCC vs. adenocarcinoma)	0.75 (0.51–1.08)	0.120	–	–
ECOG ≥ 2 vs. ECOG 0–1	1.38 (0.90–2.12)	0.138	–	–
PD-L1 1–49% vs. <1%	0.90 (0.57–1.43)	0.666	0.91 (0.57–1.45)	0.689
PD-L1 ≥ 50% vs. <1%	0.57 (0.34–0.96)	0.034	0.61 (0.36–1.01)	0.056
Current smoker vs. never smoker	1.13 (0.60–2.12)	0.704	–	–
Former smoker vs. never smoker	1.31 (0.64–2.65)	0.449	–	–
PPI use vs. no PPI use	2.01 (1.39–2.89)	<0.001	2.22 (1.52–3.24)	<0.001
Metformin use vs. no metformin use	0.79 (0.50–1.25)	0.320	–	–
Statin use vs. no statin use	0.96 (0.63–1.47)	0.864	–	–
NSAID use vs. no NSAID use	1.04 (0.73–1.49)	0.807	–	–

Abbreviations: PFS, progression-free survival; HR, hazard ratio; CI, confidence interval; ECOG, Eastern Cooperative Oncology Group; SCC, squamous cell carcinoma; PD-L1, programmed death-ligand 1; PPI, proton pump inhibitor; NSAID, nonsteroidal anti-inflammatory drug. Note: Variables with *p* < 0.10 in univariable analysis were included in the multivariable Cox regression model.

**Table 4:** Cox Regression Analysis for Overall Survival (OS).

Variable	Univariable HR (95% CI)	*p*-Value	Multivariable HR (95% CI)	*p*-Value
Age, per 1-year increase	0.996 (0.971–1.022)	0.776	–	–
Male vs. female	0.53 (0.33–0.85)	0.008	0.41 (0.25–0.69)	0.001
Histology (SCC vs. adenocarcinoma)	0.79 (0.51–1.20)	0.272	–	–
ECOG ≥ 2 vs. ECOG 0–1	2.09 (1.33–3.28)	0.001	2.21 (1.39–3.51)	0.001
PD-L1 1–49% vs. <1%	1.16 (0.66–2.02)	0.599	1.34 (0.76–2.38)	0.312
PD-L1 ≥ 50% vs. <1%	0.56 (0.31–1.03)	0.062	0.70 (0.38–1.28)	0.245
Current smoker vs. never smoker	1.25 (0.57–2.74)	0.576	–	–
Former smoker vs. never smoker	1.70 (0.73–3.93)	0.213	–	–
PPI use vs. no PPI use	1.53 (1.01–2.30)	0.043	2.01 (1.28–3.15)	0.002
Metformin use vs. no metformin use	0.78 (0.47–1.31)	0.356	–	–
Statin use vs. no statin use	1.14 (0.71–1.83)	0.580	–	–
NSAID use vs. no NSAID use	1.08 (0.72–1.62)	0.710	–	–

Abbreviations: OS, overall survival; HR, hazard ratio; CI, confidence interval; ECOG, Eastern Cooperative Oncology Group; SCC, squamous cell carcinoma; PD-L1, programmed death-ligand 1; PPI, proton pump inhibitor; NSAID, nonsteroidal anti-inflammatory drug. Note: Variables with *p* < 0.10 in univariable analysis were included in the multivariable Cox regression model.

**Table 5:** Kaplan–Meier Survival Analysis According to the Four Studied Medications.

Medication	Median PFS, Months (95% CI)	*p*-Value	Median OS, Months (95% CI)	*p*-Value
PPI use		<0.001		0.036
No	9 (7.6–10.4)		14 (8.5–19.5)	
Yes	5 (4.2–5.8)		9 (6.5–11.5)	
Metformin use		0.288		0.340
No	6 (4.9–7.1)		11 (8.5–13.5)	
Yes	8 (5.1–10.9)		15 (9.9–20.1)	
Statin use		0.855		0.568
No	7 (6.0–8.0)		14 (12.0–16.0)	
Yes	6 (3.3–8.7)		11 (8.5–13.5)	
NSAID use		0.656		0.349
No	8 (6.9–9.1)		12 (9.8–14.2)	
Yes	6 (4.6–7.4)		14 (10.0–18.0)	

Abbreviations: CI, confidence interval; NSAID, nonsteroidal anti-inflammatory drug; OS, overall survival; PFS, progression-free survival; PPI, proton pump inhibitor. Note: Survival estimates were calculated using the Kaplan–Meier method and compared using the log-rank test.

## Data Availability

The data presented in this study are available on request from the corresponding author due to patient privacy and ethical restrictions.

## Supplementary Materials

The following supporting information can be downloaded at: https://www.mdpi.com/article/10.3390/medicina62071413/s1, Table S1: Sensitivity multivariable Cox regression analysis including all concomitant medications for PFS; Table S2: Sensitivity multivariable Cox regression analysis including all concomitant medications for OS; Table S3: Sensitivity multivariable Cox regression analysis for PFS after excluding eight patients who initiated PPI treatment after nivolumab initiation; Table S4: Sensitivity multivariable Cox regression analysis for OS after excluding eight patients who initiated PPI treatment after nivolumab initiation.

## Abbreviations

The following abbreviations are used in this manuscript:

CI	Confidence Interval
ECOG	Eastern Cooperative Oncology Group
HR	Hazard Ratio
ICI	Immune Checkpoint Inhibitor
NSAID	Nonsteroidal Anti-inflammatory Drug
NSCLC	Non-Small-Cell Lung Cancer
OS	Overall Survival
PD-L1	Programmed Death-Ligand 1
PFS	Progression-Free Survival
PPI	Proton Pump Inhibitor
SPSS	Statistical Package for the Social Sciences
