# Supplementary material for: Proton Pump Inhibitor Use and Survival Outcomes in Patients with Advanced Non-Small-Cell Lung Cancer Receiving Immunotherapy: A Real-World Study
Source: Medicina (Kaunas). 2026 Jul 21;62(7):1413. doi: 10.3390/medicina62071413 (PMC13413673; doi:10.3390/medicina62071413)
Supplement: Supplementary file 1 [file medicina-62-01413-s001.zip › medicina-4437341-supplementary.pdf]

Supplementary Table S1. Sensitivity multivariable Cox regression analysis including all concomitant medications for PFS

Variable	HR (95% CI)	p-value
NSAID use vs. no NSAID use	1.05 (0.71–1.56)	0.819
Metformin use vs. no metformin use	0.70 (0.42–1.15)	0.157
Statin use vs. no statin use	0.91 (0.57–1.46)	0.694
PPI use vs. no PPI use	2.40 (1.61–3.60)	<0.001
ECOG ≥2 vs. ECOG 0–1	1.42 (0.91–2.22)	0.123
PD-L1 1–49% vs. <1%	0.90 (0.56–1.46)	0.679
PD-L1 ≥50% vs. <1%	0.58 (0.34–0.98)	0.042
Male vs. female	0.59 (0.37–0.93)	0.023

Abbreviations: PFS, progression-free survival; HR, hazard ratio; CI, confidence interval; ECOG, Eastern Cooperative Oncology Group; PD-L1, programmed death-ligand 1; PPI, proton pump inhibitor; NSAID, nonsteroidal anti-inflammatory drug

Note: Variables with p<0.10 in univariable analysis were included in the primary multivariable model. In addition, a sensitivity multivariable Cox regression model including all concomitant medications (NSAIDs, metformin, statins, and PPIs) was constructed regardless of univariable significance.

Supplementary Table S2. Sensitivity multivariable Cox regression analysis including all concomitant medications for OS

Variable	HR (95% CI)	p-value
NSAID use vs. no NSAID use	1.02 (0.65–1.60)	0.935
Metformin use vs. no metformin use	0.71 (0.41–1.21)	0.207
Statin use vs. no statin use	1.33 (0.80–2.20)	0.266
PPI use vs. no PPI use	1.97 (1.23–3.16)	0.005
ECOG ≥2 vs. ECOG 0–1	2.14 (1.34–3.42)	0.001
PD-L1 1–49% vs. <1%	1.28 (0.72–2.27)	0.403
PD-L1 ≥50% vs. <1%	0.67 (0.37–1.24)	0.203
Male vs. female	0.41 (0.24–0.68)	0.001

Abbreviations: OS, overall survival; HR, hazard ratio; CI, confidence interval; ECOG, Eastern Cooperative Oncology Group; PD-L1, programmed death-ligand 1; PPI, proton pump inhibitor; NSAID, nonsteroidal anti-inflammatory drug.

Note: Variables with p<0.10 in univariable analysis were included in the primary multivariable model. In addition, a sensitivity multivariable Cox regression model including all concomitant medications (NSAIDs, metformin, statins, and PPIs) was constructed regardless of univariable significance.

Supplementary Table S3. Sensitivity multivariable Cox regression analysis for PFS after excluding eight patients who initiated PPI treatment after nivolumab initiation

Variable	HR (95% CI)	p-value
NSAID use vs. no NSAID use	1.16 (0.80–1.69)	0.441
Metformin use vs. no metformin use	0.63 (0.38–1.06)	0.084
Statin use vs. no statin use	0.83 (0.51–1.35)	0.443
PPI use vs. no PPI use	2.47 (1.65–3.71)	<0.001
ECOG ≥2 vs. ECOG 0–1	1.43 (0.90–2.26)	0.131
PD-L1 1–49% vs. <1%	0.96 (0.59–1.57)	0.873
PD-L1 ≥50% vs. <1%	0.51 (0.29–0.91)	0.022
Male vs. female	0.59 (0.37–0.93)	0.023

Abbreviations: PFS, progression-free survival; HR, hazard ratio; CI, confidence interval; ECOG, Eastern Cooperative Oncology Group; PD-L1, programmed death-ligand 1; PPI, proton pump inhibitor; NSAID, nonsteroidal anti-inflammatory drug.
Note: This sensitivity analysis excluded eight patients who initiated PPI treatment after nivolumab initiation.

Supplementary Table S4. Sensitivity multivariable Cox regression analysis for OS after excluding eight patients who initiated PPI treatment after nivolumab initiation

Variable	HR (95% CI)	p-value
NSAID use vs. no NSAID use	0.79 (0.50–1.23)	0.291
Metformin use vs. no metformin use	0.71 (0.41–1.24)	0.227
Statin use vs. no statin use	1.38 (0.81–2.35)	0.231
PPI use vs. no PPI use	1.91 (1.20–3.05)	0.007
ECOG ≥2 vs. ECOG 0–1	2.06 (1.28–3.34)	0.003
PD-L1 1–49% vs. <1%	1.34 (0.73–2.45)	0.344
PD-L1 ≥50% vs. <1%	0.70 (0.36–1.36)	0.288
Male vs. female	0.40 (0.24–0.68)	0.001

Abbreviations: OS, overall survival; HR, hazard ratio; CI, confidence interval; ECOG, Eastern Cooperative Oncology Group; PD-L1, programmed death-ligand 1; PPI, proton pump inhibitor; NSAID, nonsteroidal anti-inflammatory drug.
Note: This sensitivity analysis excluded eight patients who initiated PPI treatment after nivolumab initiation.
